# Quantifying preference for social stimuli in young children using two tasks on a mobile platform

**DOI:** 10.1371/journal.pone.0265587

**Published:** 2022-06-01

**Authors:** Indu Dubey, Simon Brett, Liliana Ruta, Rahul Bishain, Sharat Chandran, Supriya Bhavnani, Matthew K. Belmonte, Georgia Lockwood Estrin, Mark Johnson, Teodora Gliga, Bhismadev Chakrabarti, on behalf of the START consortium

**Affiliations:** 1 Centre for Autism, School of Psychology & Clinical Language Sciences, University of Reading, Reading, United Kingdom; 2 Institute of Applied Sciences and Intelligent Systems, National Research Council of Italy (CNR), Messina, Italy; 3 Department of Computer Science and Engineering, Indian Institute of Technology Bombay, Mumbai, India; 4 Centre for Chronic Conditions and Injuries, Public Health Foundation of India, Gurgaon, India; 5 Sangath, New Delhi, India; 6 Com DEALL Trust, Bangalore, India; 7 Division of Psychology, Nottingham Trent University, Nottingham, United Kingdom; 8 Centre for Brain and Cognitive Development, Birkbeck University of London, London, United Kingdom; 9 School of Psychology, University of Cambridge, Cambridge, United Kingdom; 10 School of Psychology, University of East Anglia, Norwich, United Kingdom; 11 Department of Psychology, Ashoka University, Sonipat, India; 12 India Autism Center, Kolkata, India; University of Iowa, UNITED STATES

## Abstract

Children typically prefer to attend to social stimuli (e.g. faces, smiles) over non-social stimuli (e.g. natural scene, household objects). This preference for social stimuli is believed to be an essential building block for later social skills and healthy social development. Preference for social stimuli are typically measured using either passive viewing or instrumental choice paradigms, but not both. Since these paradigms likely tap into different mechanisms, the current study addresses this gap by administering both of these paradigms on an overlapping sample. In this study, we use a preferential looking task and an instrumental choice task to measure preference for social stimuli in 3–9 year old typically developing children. Children spent longer looking at social stimuli in the preferential looking task but did not show a similar preference for social rewards on the instrumental choice task. Task performance in these two paradigms were not correlated. Social skills were found to be positively related to the preference for social rewards on the choice task. This study points to putatively different mechanisms underlying the preference for social stimuli, and highlights the importance of choice of paradigms in measuring this construct.

## Introduction

Social interactions are one of the core components of our daily lives. Neurotypical infants make attempts to engage in social interaction from as early as 6–8 weeks of age, responding to their caregivers by smiling or cooing [1]. Children spend longer looking at faces than at comparable non-social stimuli with matched spatial frequency [2], they prefer biological to non-biological motion [3], and smile more in response to social than non-social stimuli [4]. These studies indicate high social reward responsivity in early years of life. This bias for social stimuli is also shaped by contextual parameters such as familiarity[5].

While there are several paradigms to evaluate social reward responsivity (47), we focus on two most commonly used classes of paradigms used to study this construct. One class consists of passive paradigms that present the social stimulus (e.g. a smiling face) and measure the pleasure/consummatory aspect of the participant’s response. These measures can be observational (e.g. gaze duration in an eye-tracking measure, or reward-related neural response in a neuroimaging measure), or self-reported (e.g. ‘rate how much you like this’). In contrast, the second class of paradigms are instrumental tasks that present conventionally rewarding social stimuli contingent on the performance of a particular behaviour (typically a motor act such as pressing buttons). In these tasks, the choice behaviour as indexed through the number or speed of the responses is taken to provide a measure of social reward responsivity. While the former class of paradigms is related to how much children want and enjoy engaging in a social interaction, the latter is more related to whether social interaction can reinforce other behaviours. Both of these components are vital for social functioning.

While self-report studies arguably provide a subjective account of how rewarding a stimulus is, ratings and language-based tools are easily influenced by experimenter or subjective biases and are not suitable for use in populations with low verbal abilities, such as very young children [6–8]. To minimise such biases, preferential looking paradigms using camera-based or continuous eye-tracking technology have been used to index relative reward value [9, 10]. These studies are based on the assumption that longer gaze duration toward a specific set of stimuli indicates greater interest and greater positive value for those stimuli [10–12]. In the context of this study, we only discuss preferential looking paradigms as used to assess relative preference between different rewarding stimuli. In a typical preferential looking paradigm for social versus non-social rewards, two competing stimuli- one of each type- are simultaneously presented while participants’ gaze is tracked [13, 14]. These studies have demonstrated that neurotypical children and adults gaze longer at social than non-social stimuli [14–17]. Children as well as adults thus demonstrate a robust preference for social rewards, when measured using visual preference paradigms.

Comparatively fewer studies have used an instrumental task to test preference for social stimuli. One of the early attempts at addressing this gap was the Social Incentive Delay task (SID), in which participants are aware of the reward (social/non-social) they would receive at the end of each trial on a *reaction time* game [18]. Here, reaction time in anticipation of social reward is used as a measure of social reward responsivity. Neurotypical adults react faster to monetary rewards than social (smiling faces) rewards [18]. Another study using a similar paradigm compared two age groups of younger adults (20–28 years) and older adults (60–78 years), both of whose hit rates (accuracies), were significantly higher for monetary than social (smiling faces) rewards [18, 19]. The same study found that the reaction time was influenced by the reward magnitude and not the type (social vs. monetary), thus providing no evidence of preference for social stimuli. The second set of studies examining social reward responsivity in instrumental paradigms used *effort* as an index of social reward responsivity. In tasks of this type, participants exert different amounts of effort (made through button presses) to obtain a social or a non-social reward [20, 21]. These tasks demonstrate a preference for social stimuli, in as young children and neurotypical adults invest more effort to get social reward than non-social rewards. However, no such preference is seen in neurotypical adolescents [21–23]. Ruta and colleagues used a similar but simpler paradigm targeted for younger children (14–68 months), where children choose one of two buttons to look at social or non-social images [24]. They found that while neurotypical children do not show any preference for the ‘social’ stimulus, autistic children demonstrated a lower preference for social stimuli in the choice task. The authors interpreted the lack of a preference for social stimuli in neurotypical children to the nature of nonsocial stimuli used (trains) in this study. It was hypothesised that choosing a less salient nonsocial stimulus (e.g. a geometric pattern) might reveal a preference for social stimuli in neurotypical children.

In summary, studies evaluating social reward responsivity in the neurotypical population have reported mixed findings ranging from high preference for social stimuli to no preference. In paradigms where the stimuli are only passively processed, measuring consummatory behavior, participants typically show a higher preference for social stimuli. On the other hand, paradigms that involve an instrumental component do not always show this ‘social advantage’. The scope of inference has been limited by the absence of any study using both of these paradigms in the same set of individuals. The current study addresses this gap using both a preferential looking task and an instrumental choice task in young children administered on a mobile platform (a tablet PC). In line with previous lab-based studies, we hypothesise that children will show a visual preference for social stimuli in the both tasks.

## Methods

### Participants

Children between ages 3–9 years were recruited from the summer scientist week–a public science engagement event at the University of Nottingham, where parents from the local community brought in their children. Children took part in a number of experiments, each of which was conducted in a small booth within a large open space. The study used a convenience sampling method, and accordingly sample demographics e.g. age range, gender ratio, and number of participants, were influenced by the nature of the event. The preferential looking task (described in the tools section) was completed by 98 children (54 females). Their mean age was 5.73 years (SD ±1.25). The button task (described in the tools section) was completed by a partially overlapping sample of 98 children (48 females). Their mean age was 5.67 years (SD ±1.19). Due to technical errors with the app, complete data on both tasks are available on ~60% of the sample (n = 62) (30 females; mean age 5.64 SD ±1.15).

### Stimuli

The social and non-social video stimuli used in the tasks were taken from the commercial image/video sharing website www.shutterstock.com. The social stimuli included videos of one or more children smiling and looking at the camera or a parent and child smiling and interacting with each other. The non-social stimuli included videos of falling pieces of puzzles, dynamic patterns, spinning wind-fans or washing machines. List of the videos used for the tasks is given in the S1 File. Each video clip (no sounds) was trimmed to 5 seconds for both the tasks. Both tasks were presented on a tablet PC (Samsung 10.1 tablet SM P600) running the START app. Details of the app and the START project are available at the https://startproject.bhismalab.org [25].

### Tools

#### Preferential looking task

**Fig 1:**
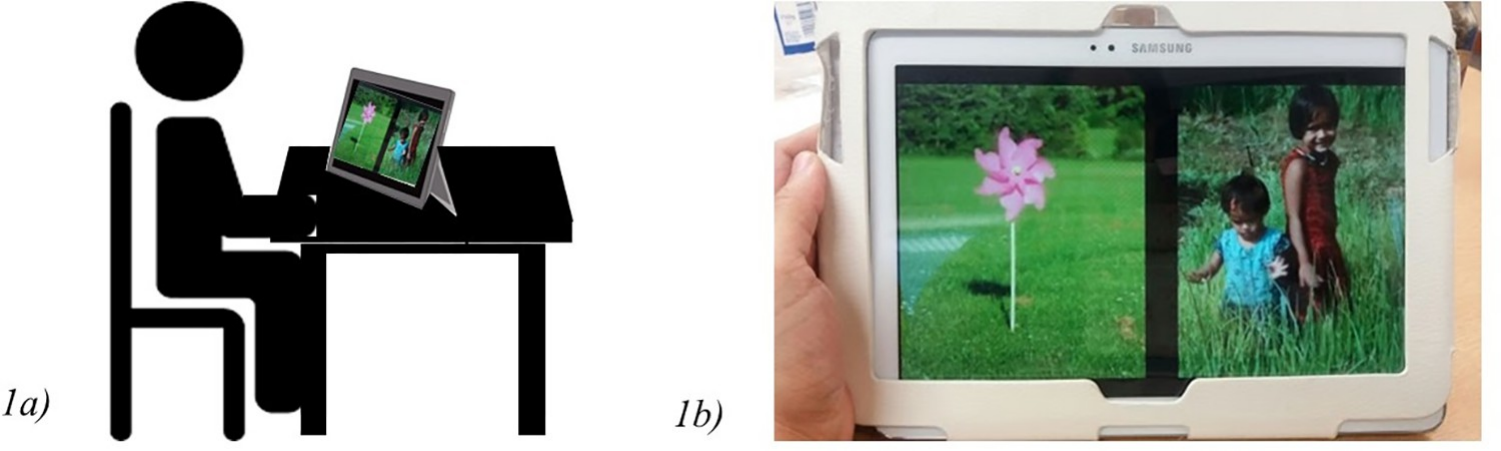
a) Schematic presentation of the setup, b) A sample trial from the preferential looking task shown on a tablet device.

This task was similar to paradigms used by Pierce and colleagues to measure preference for social stimuli in children on the autism spectrum [14, 26] (we use the phrases children/individuals on the autism spectrum and autistic children/individuals interchangeably to acknowledge the diversity of views regarding terminology within the autism community). Four pairs of social and non-social videos each presented twice in a fixed order. The side of presentation (left/right half of screen) and video type (social/non-social) was counterbalanced (Fig 1). A video demonstrating the presentation of the tasks on the tablet is available as the A1 Appendix in S1 Video. Each video covered nearly half of the screen, leaving a gap of 0.5cm between them.

#### Button task

This task was based on a similar tablet-based task used previously to measure preference for social stimuli in young children on the autism spectrum and matched controls [24]. This was an instrumental choice task in which participants were shown two “buttons” on the screen, which presented either a social or a non-social stimulus when touched. The position of the “buttons” on the screen was randomly changed in every trial to overcome any side or handedness bias. Unlike the original version of the task which used static pictures, the stimuli in this version were two sets of videos: 1) social e.g. a child swimming underwater and waving or a mother and a child hugging each other, and 2) non-social e.g.jumping balls or a geometric wave-like pattern. Videos instead of images were used to increase salience [27]. These videos were presented in full screen display after the participant touched the button to make the choice. A sample trial is presented in the Fig 2 and a video demonstration of the task is presented in the A2 Appendix in S2 Video. There were eight choice trials, for which the responses were captured using the device’s touch sensors.

**Fig 2:**
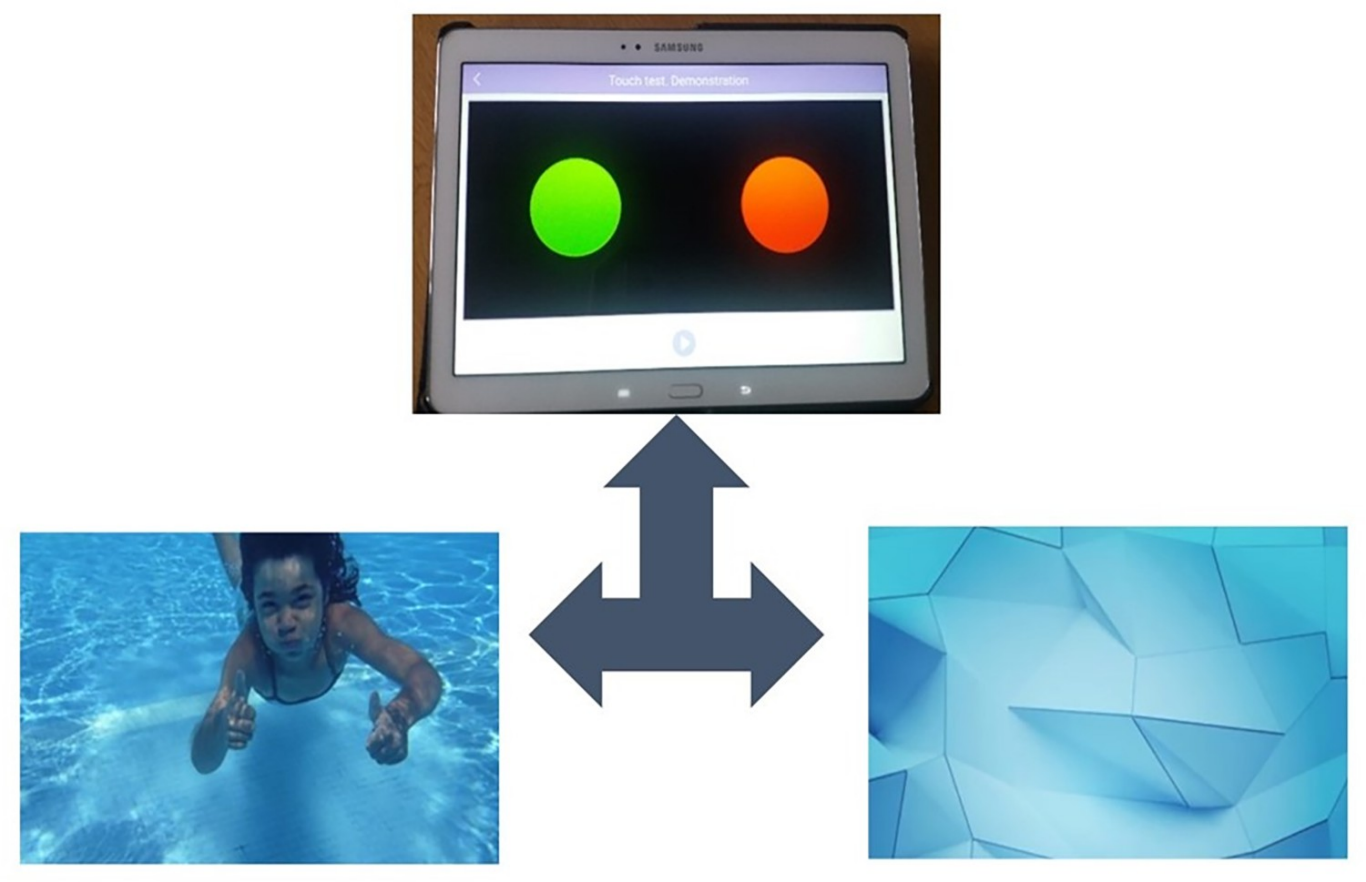
A sample trial from button task showing choice presented on the tablet screen and linked sample of videos.

#### British Picture Vocabulary Scale (BPVS) 3^rd^ edition

Verbal intelligence was measured using the British Picture Vocabulary Scale (BPVS) 3^rd^ edition [28]. This measure allowed us to test the impact of intelligence on the putative metrics of preference for social stimuli. We report the raw score on BPVS for statistical analysis.

#### Social Aptitude Scale (SAS)

The Social Aptitude Scale (SAS) is a 10-item parent report scale to evaluate social skills in children (5–16 years) [29]. This measure allows for a potential test of criterion validity in view of the suggested link between social motivation and social skills [30, 31].

### Procedure

Ethical approval for the study was provided by the ethics committee of the School of Psychology, University of Nottingham. All procedures performed in this study were in accordance with the ethical standards of the University of Nottingham and with the 1964 Helsinki declaration and its later amendment in 2008. Informed consent was obtained from the primary caretaker of all individual participants (under 18 years of age). Each participant provided verbal assent prior to taking part in the study. Participants were invited to play some “games” on a tablet in a quiet section of a large room (shared with other researchers). They sat at a child-sized table and chair and completed both tasks in a single sitting. The preferential looking task was run first, followed by the button task. It took less than 10 minutes for each child to complete both the tasks. During the public engagement event, participants were also evaluated for their verbal intelligence using BPVS and their primary caretakers completed the SAS. These scores were made available to researchers after data collection. All the children (irrespective of their performance) were given a gift pack for taking part in the public engagement event.

#### Preferential looking task

The tablet was positioned upright on a stand facing the child (see Fig 1A). The task started with a preparatory phase during which tablet’s position was adjusted to ensure a) the child’s eyes and face were visible to the front camera of the tablet, b) there was sufficient illumination, c) the face was within 35-50cm from the tablet, and d) the tablet was stable. On each trial a set of two video clips: a social and non-social were shown on the screen for five seconds, examples shown in Fig 1B and a video file available in A1 Appendix in S1 Video. The eight trials continued without any gap or central fixation cue and the participants were encouraged to stay still while looking at the screen.

#### Button task

The task was presented with the tablet placed flat on the table. It began with a demonstration phase showing the two ‘buttons’ present at the beginning of every trial. The experimenter then verbally instructed and demonstrated to the children that one of these buttons had videos of ‘people’ while the other had the videos of ‘things’ (see example Fig 2 and sample video of the task in A2 Appendix in S2 Video). Participants were then asked to touch the two buttons and observe examples of stimuli, to ensure task comprehension. They were then shown eight experimental trials in which they were free to choose to touch any button to look at the social or non-social videos. There was no time limit for responding.

### Data processing

#### Preferential looking task

The face and eyes from the video recording using the front camera of the tablet were automatically extracted while the participants looked at the screen during the task. This video feed was then processed using an adapted version of a gaze estimation algorithm [32, 33]. The algorithm utilizes a deep neural network model trained on the *gazecapture* database. The database is a corpus of 2.5 million images collated from 1474 subjects. The original model is shown to have achieved state of the art results with 2.53 cm prediction error on iOS-based tablet devices. We observed that the model was not trained on children of the requisite age group, and neither have the experiments been conducted in a casual setting on Android-based devices. We therefore re-validated our implementation of the algorithm on data collected from children performing the preferential looking task by manually annotating 4952 frames extracted from the videos of 8 children. We observed a 91.23% accuracy in validation using this approach. Here, accuracy refers to the percentage of frames correctly labeled by the algorithm as compared to the manual labels. For the statistical test reported below, the percentage of gaze on social stimuli was calculated as follows:

Percentage of gaze on social stimuli =

{N(frames with gaze on social stimuli)/N(total frames with gaze on social or non-social stimuli)}*100

#### Button task

Proportion of trials on which participant chose the button linked with the social stimulus was calculated for each participant as an index of preference for social stimuli.

Preference for social stimuli =

{N(trials on which social stimulus was chosen)/N(total trials completed)}*100

### Statistical analyses

Wilcoxon sign rank test against a chance value of 50 was run to ascertain any significant preference for social stimuli on both the tasks. A linear regression was run with social skill measure (SAS), gender, age, and an interaction between age & gender as predictors of preference for social stimuli on the percentage of gaze on social stimuli in the preferential looking task. An ordinal logistic regression was run with social skill measure (SAS), gender, age, and an interaction between age & gender as predictors of preference for social stimuli on the button task. The association between preference for social stimuli as measured on the two tasks was tested using a correlation analysis. Since verbal intelligence is closely linked with social skills [34, 35] we do not include BPVS scores in the regression models. We report the relationship between BPVS and measures of preference for social stimuli using partial correlation, controlling for the effects of age and gender.

The main analyses were rerun on the subset of participants who completed both tasks, and on the subset of participants aged > = 5 year, for completeness. These results are reported in the (S3 and S4 Sections in S1 File).

All statistical analyses were conducted in Jamovi v 1.6.23 [36].

## Results

An outlier check was done for both sets of data using Cook’s distance taking cut off as 4/n. This identified seven potential outliers for the preferential looking task and nine potential outliers for the button task. Statistical analyses were run without these participants for both the tasks. We found that exclusion of any of these data points did not change the findings, so we report the results with the full sample including all the participants.

### Preferential looking task

**Fig 3:**
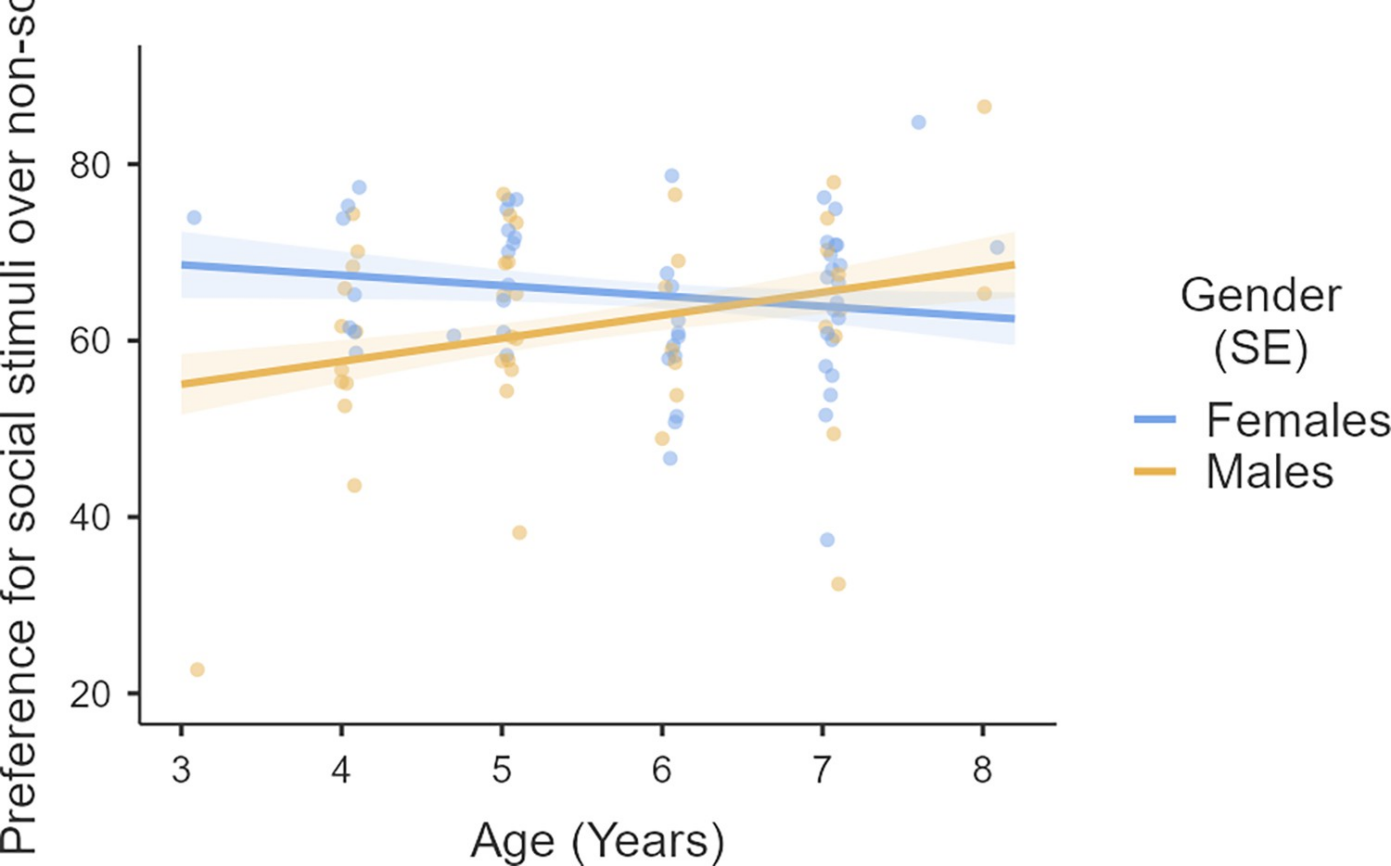
Line graph shows the interaction between age and gender when predicting preference for social stimuli on preferential looking task.

The results from this task suggest that participants looked towards the social stimuli more than expected by chance (*W* (98) = 4588, *p <* .*001*, *M* = 63.56, *SD* = 10.55). The results of the linear regression indicated that the gmodel explained 11.3% of the variance. SAS (*β* = 0.033, *p* = .753) and age (*β* = -0.147, *p* = .293) of participants were not significant predictors. However, gender (*β* = -0.414, *p* = .011) and an interaction between gender and age (*β* = 0.478, *p* = .03) significantly predicted preference for social stimuli. Fig 3 illustrates the preference for social stimuli in male and female participants over age. To understand these results better we divided the participants into two age bins: younger [3–6 years] (f = 20, m = 26) and older [6.1- >9 years] (f = 34, m = 18) participants. We compared the preference for social stimuli between males and females in these two subgroups. The results show that younger females have a stronger preference for social stimuli compared to males (U = 137, p = .006) but this gender difference is not seen in the older participants (U = 301, p = 0.932).

The proportion of gaze on social stimuli and verbal intelligence (BPVS raw score) (*r*_s_ = .070, n = 87, *p =* .*524*) were not significantly correlated when controlled for the effects of age and gender.

### Button task

**Fig 4:**
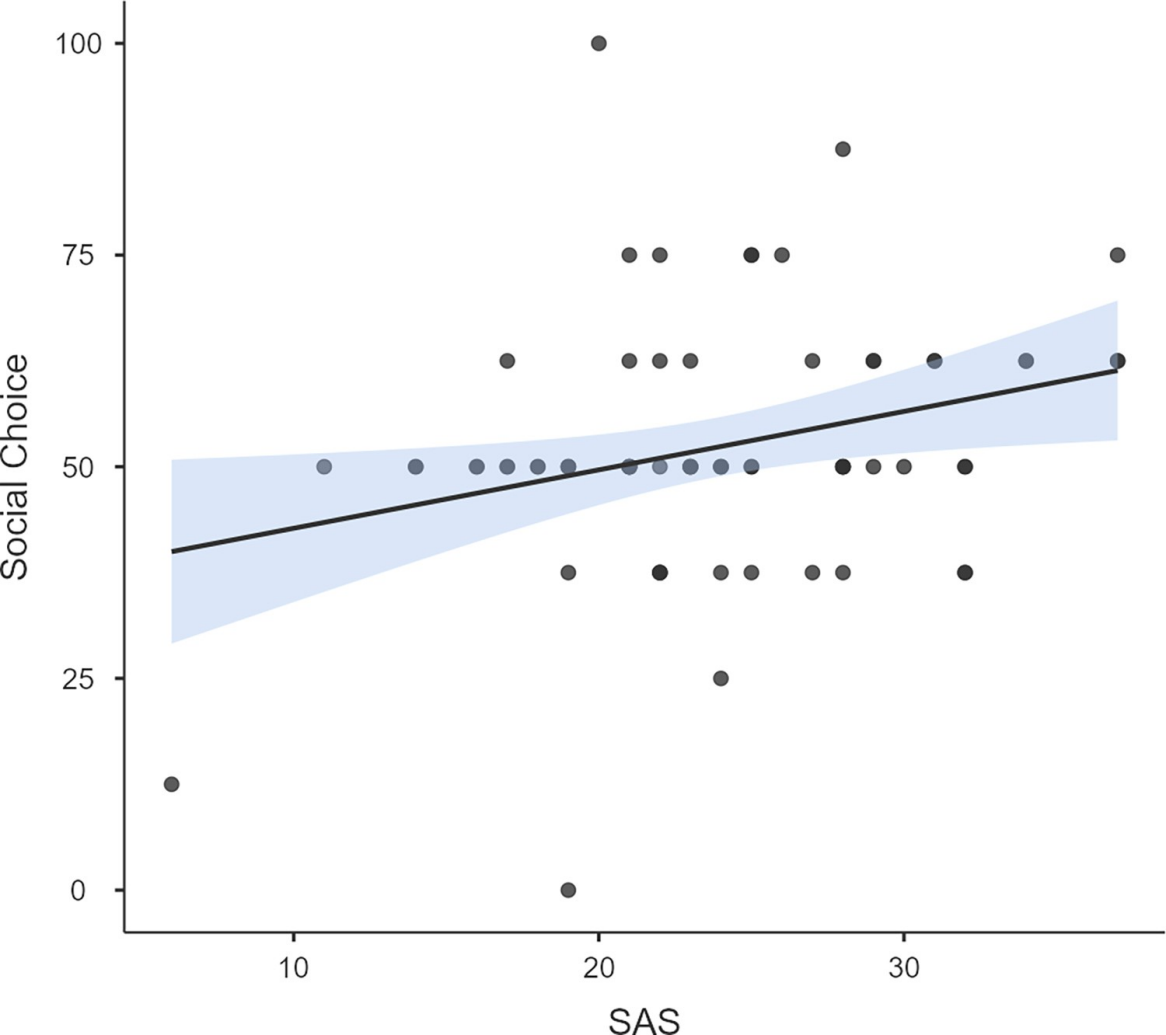
Scatter graph for social skills as measured on SAS and the preference for social stimuli as measured on button task (for participants above age 5 years).

Participants did not choose the button for social stimuli more than expected by chance (*W* = 858, *p =* .*3*, *M* = 51.3, *SD* = 16). Ordinal logistic regression revealed that score on SAS significantly predicted the choice for social stimuli on this task (Z = 2.6, p = 0.009) (Fig 4). Neither age (Z = -.161, p = .872), gender (Z = -.714, p = .475), nor their interaction (Z = .424, p = .671*)* significantly predicted the choice for social stimuli. There was no significant correlation between the preference for social stimuli and verbal intelligence (BPVS raw) (*r*_s_ = —.031, n = 85, *p =* .*782*) after partialling out the effects of age and gender.

### Inter-task comparison

Preferences for social stimuli across the two tasks: Preferential looking and Button tasks (completed by 62 participants) were not correlated with each other (*r*_s_ = .15, *p =* .*246*).

## Discussion

This study evaluated two putative task measures of social reward responsivity in typically developing children using a tablet PC. We found a preference for social over non-social stimuli in a preferential looking task, replicating results from similar studies using lab-based eye-trackers [15, 16]. This preference for social stimuli was not seen in the instrumental choice (button) task. Despite this absence of overall preference for social stimuli in the button task, a positive relationship was noted between children’s social skills and their preference to choose the button associated with the social stimulus, replicating previous results. Interestingly, these two measures of preference for social stimuli were uncorrelated in this dataset, pointing to the possibility that these tasks index distinct processes within the broader construct of social reward responsivity. We discuss these results below, highlighting the importance of task choice in measuring social reward responsivity and its potential impact.

Studies evaluating visual preference for social stimuli in neurotypical children suggest that they spend longer looking at social stimuli [16, 17, 26, 37, 38]. It is also suggested that the more complex social stimuli (depicting multiple individuals), when used in a preferential looking task, are likely to elicit greater preference for social stimuli [39–41]. In the present study, we report similar results showing preference for social stimuli in neurotypical children using a tablet-based preferential looking task. A significant gender by age interaction effect was noted, with younger females showing greater preferential attention to social over non-social stimuli. This result mirrors previous findings of higher preference for social stimuli in females at both behavioural and neural levels [42, 43]. Similar effects of age (but not gender) on social reward responsivity has also been reported in another study suggesting stronger preference for social stimuli in younger (4–8 year old) participants compared to older children (9–12 year) [23].

Unlike preferential looking measures, behavioural approach or instrumental choice tasks have been less consistent in indicating preference for social stimuli in neurotypical children [21–24]. In the present study, we replicated the findings by Ruta and colleagues [24] using an adapted version of the original task. Children showed no significant preference for social stimuli on the button task. This observation suggests that the results by Ruta and colleagues cannot be explained fully by the choice of trains as the nonsocial stimuli. Interestingly however, a reduced preference for social stimulus was associated with low scores on a measure of social skills (SAS). Responses to social rewards have been suggested to be linked to social skills [30, 31]. This result is also consistent with the earlier study which found a reduced preference for social rewards in autistic children, who typically score low in SAS [29].

Even though the button task is a simpler version of the instrumental tasks used previously, it still requires participants to remember the association between the cue (button) and reward (linked videos). Furthermore, this task’s requirement to make motor movements to seek the social reward imposes additional demands. Thus, performance in the button task is likely to tap into processes beyond those engaged solely in reward processing. Preferential looking tasks, on the other hand, impose few/no additional demands and hence might be more directly related to consummatory or hedonic preference for social stimuli. While both these tasks seem to evaluate the common concept of social reward responsivity, it is possible that they may index distinct processes. Importantly, both the preferential looking as well as the button task for measuring social reward responsivity were administered on an overlapping sample of children, making it possible to test their inter-relationship. Interestingly, we did not find evidence for a significant relationship between the preference for social stimuli as quantified by these two tasks. While any interpretation of the lack of a relationship should be done with caution, this result supports the possibility that these two tasks tap into distinct aspects of social reward processing. Similar dissociations in different aspects of reward processing–such as wanting and liking, have been reported for other stimuli such as food or drugs, and these have been linked with atypical behaviour [44, 45]. Dissociations have also been reported between self-report and behavioural measures of social reward responsivity in clinical populations [46, 47]. Accounts of reduced social reward responsivity in clinical populations may therefore benefit from a more rigorous task-based assessment of putatively distinct components of reward processing in future studies.

Several limitations arise from the constraint of implementing these tasks in a new, mobile computing environment. First, the order of the two tasks was not counterbalanced: the preferential looking task was always run first, followed by the button task. However, it is unlikely that the fixed order of administering the two tasks would have influenced the results. Since the preferential looking task presented both social and nonsocial stimuli simultaneously, participants are not likely to experience selective fatigue with one of these two categories of stimuli. Accordingly, their behaviour on the button task is not likely to be biased toward social or nonsocial stimuli due to having completed the preferential looking task earlier. Second, the order of presentation of stimuli in the preferential looking task was non-random (social stimuli on the left in the first four trials and on the right in the next four trials). While this fixed order of counterbalanced stimuli should not influence the inferences drawn, we have fully randomised the order of presentation in a later study–and demonstrated similar results [35]. Third, in the instrumental task the association between button colour and stimulus type (social/non-social) was not counterbalanced across participants. Since the participants does not show any difference in the rates of pressing either button, it is unlikely to be a confound for the current study. However, we have added this feature in the later version of the task, to guard against confounds with baseline colour preferences [35]. Fourth, there was no memory check at the end of the button task to test if children remembered the association between the colour of the button and the type of stimulus (social/nonsocial). It is therefore possible that some participants may have forgotten the association, thus increasing noise in the data. Therefore, we recommend the use of a formal memory check at the end of similar tasks that rely on pairing between stimuli. Finally, while we administered both the preferential looking and the button task on all participants, technical errors resulted in a significant loss of data, resulting in >60% of the sample with data on both tasks. However, we find no reason to expect any of these limitations to change the interpretation. The sample of the children included in the study were recruited from an event held in the university for introducing scientific concepts to young children. It is possible that such events may be attended more by the children and families that are from higher educational backgrounds. It will be important to see if the findings can be replicated with a representative sample collected from the community.

## Conclusion

The present study reports data from two tasks that evaluate two complementary aspects of social reward responsivity, in young children. The results highlight the importance of using a range of measures to tap these different components of social reward processing. These complementary measures of social reward responsivity can provide a theoretically informed approach to stratification within and across neurodevelopmental conditions.

## Supporting information

S1 File (DOCX)

S1 Video (ZIP)

S2 Video (ZIP)
