# Supplementary material for: Quantifying preference for social stimuli in young children using two tasks on a mobile platform
Source: PLoS One. 2022 Jun 1;17(6):e0265587. doi: 10.1371/journal.pone.0265587 (PMC9159616; doi:10.1371/journal.pone.0265587)
Supplement: S1 File — (DOCX) [file pone.0265587.s001.docx]

**Supplementary Material**

**Section S1:** List of the stimuli video downloaded from shutterstock website to be used in the preferential looking and button tasks. Videos can be viewed on <https://www.shutterstock.com/video> by typing the file numbers below.

**Non-social :**

Preferential Looking:

1105690, 10815839, 10240904, 2555141

Button Task:

2628521, 3147205

**Social:**

Preferential Looking:

6218771, 10642664, 11034134, 23390677, 3410639

Button Task:

3795266, 4656107

**Section S2:** Age distribution for the three sets of data

1.
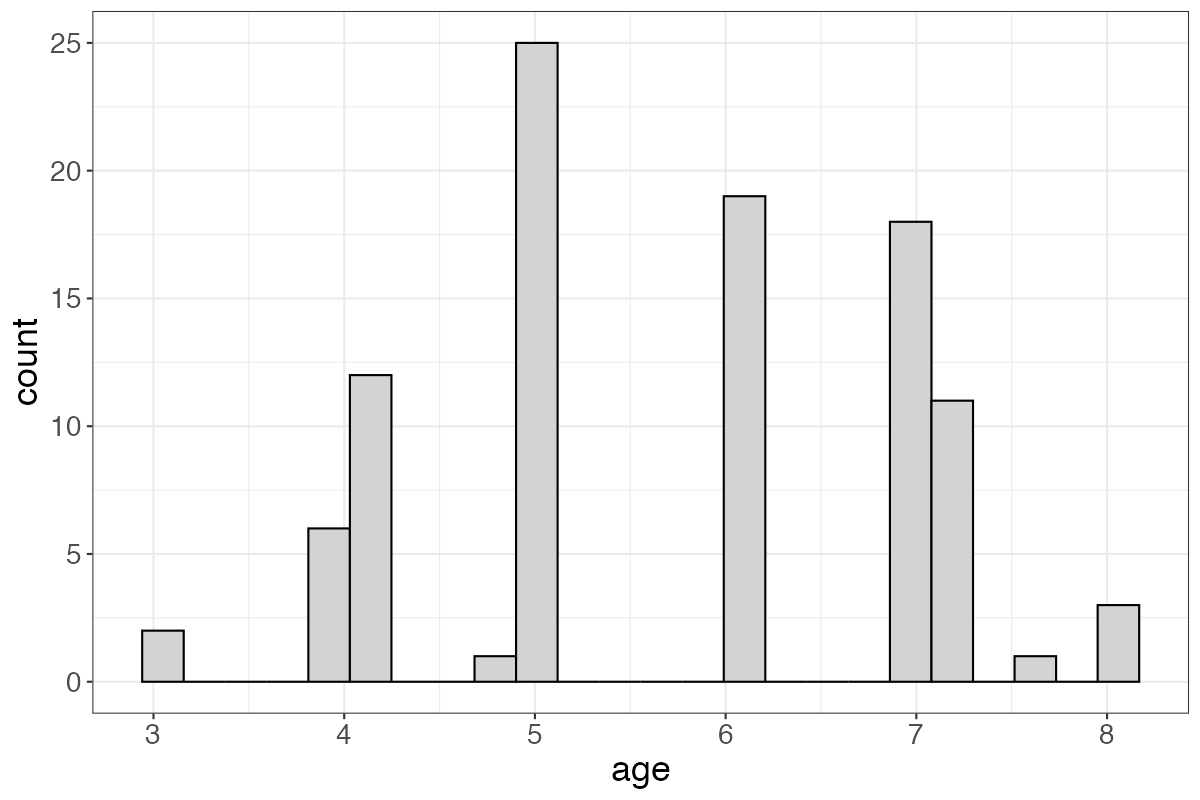
b)
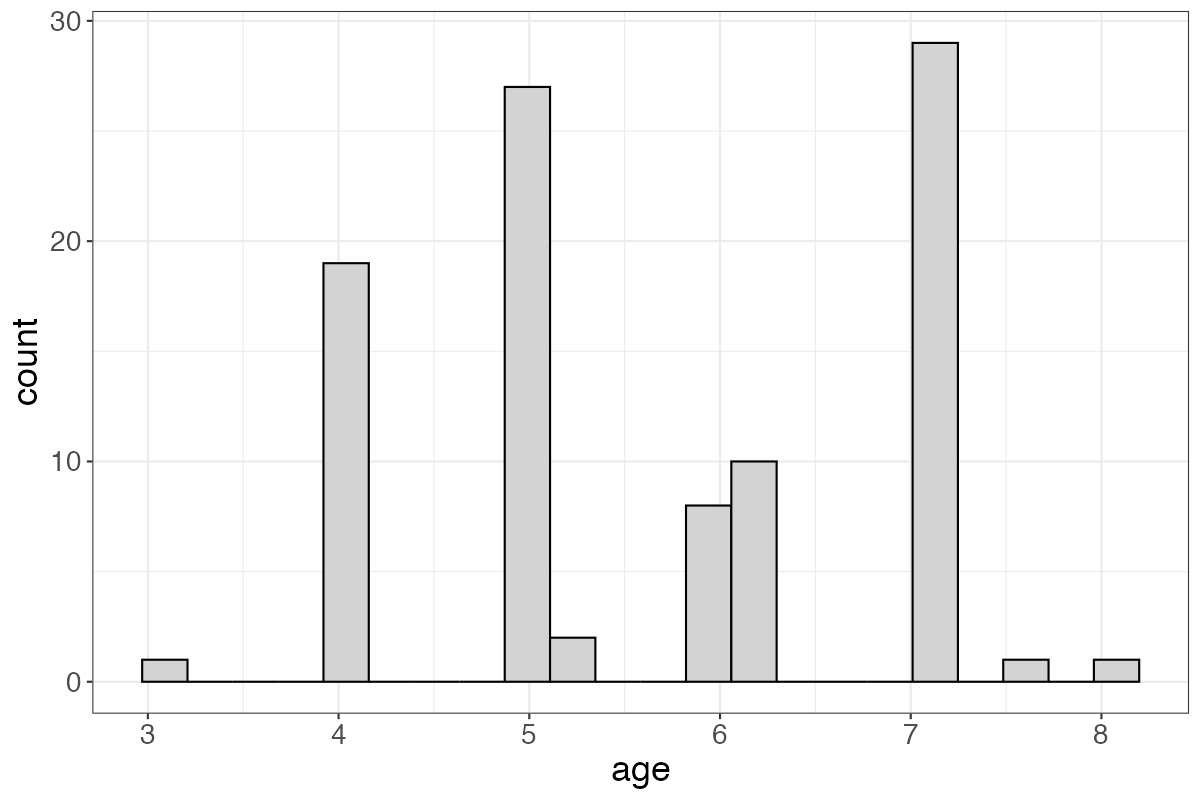


c)
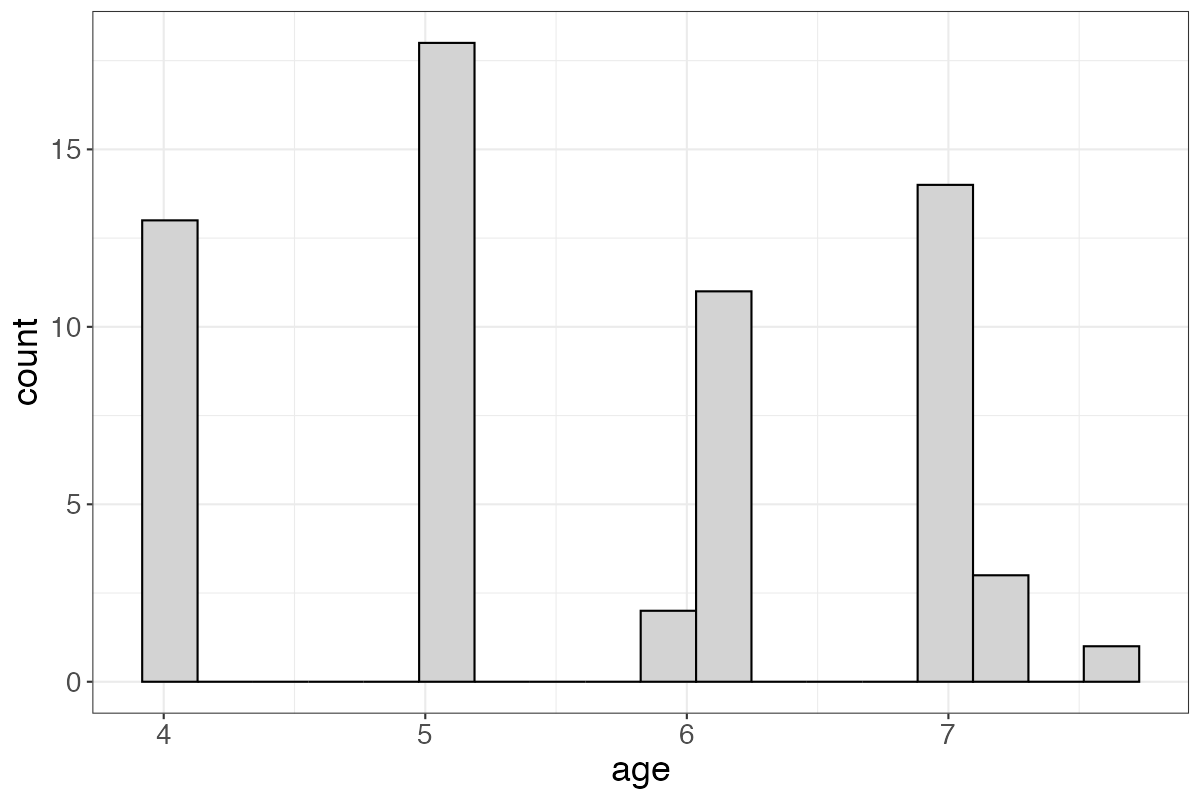


**Figure S2:** Age distribution for a) Preferential looking sample, b) Button task sample, c) Overlapping sample of participants.

Children under age 5 years: 21.42% of participants in the sample who completed preferential looking task, 20.20% of button task, and 20.96% of the subsample of those who completed both tasks were below the age of 5 years.

**Section S3:** Results on the subset of participants on whom data was available for both tasks

**Preferential Looking task**: The results from this task suggest that participants looked towards the social stimuli more than expected by chance (*W* (62) = 1877, *p* < .001, *M* = 63.4, *SD* = 9.74). The results of the linear regression indicated that the model explained 9.4% of the variance. SAS, age, gender, or the interaction between age and gender did not predict preference for social stimuli. There was no significant correlation between the proportion of gaze on social stimuli and verbal intelligence (BPVS raw score) (*r_s_* = .151, *n* = 54, *p* = .285) after controlling for the effects of age and gender.

**Button task:** Participants did not choose the button for social stimuli more than expected by chance (*W (62)*=375, *p* = .171, *M* = 52.6, *SD* = 16.2). Ordinal logistic regression results show that SAS, age, gender, or the interaction between age and gender did not predict preference for social stimuli. There was no significant correlation between the preference for social stimuli and verbal intelligence (BPVS raw) (*r_s_* = .004, *n* = 54, *p* = .975) after partialling out the effects of age and gender.

**Section S4:** Results on each task for all participants aged 5-9 years

Participants below the age of 5 years (21.42% of the sample for preferential looking task, 20.20% of the sample for button task, and 20.96% of the sample which completed both tasks) could not be included in some analyses. Here we present the analyses on data excluding these participants i.e. data collected from participants between age 5-9 years only.

**Preferential looking task:** After excluding this subset of participants the remaining sample had *n* = 77 and the data were normally distributed. The results from one-sample t test against the test value of 50 suggest that participants looked towards the social stimuli more than expected by chance (*t* (76) = 12.2, *p* < .001, *M* = 64, *SD* =10.1). The results of the linear regression indicated that the model explained 7.2% of the variance. SAS, age, gender, or the interaction between age and gender did not predict preference for social stimuli. There was no significant correlation between the proportion of gaze on social stimuli and verbal intelligence (BPVS raw score) (*r_s_* = .125, *n* = 71, *p* = .307) after controlling for the effects of age and gender.

**Button task:** Participants did not choose the button for social stimuli more than expected by chance (*W* = 481, *p* = .328, *n* = 78, *M* =51.4, *SD* =16.6). Ordinal logistic regression results show that SAS scores predicted preference for social stimuli (Z=2.627, p=.009) but age (*Z=-.353, p=.724*), gender (*Z=.171, p=.864*), nor the interaction between two (*Z=-.34, p=.734)* were significant predictors. There was no significant correlation between the preference for social stimuli and verbal intelligence (BPVS raw) (*r_s_* = -0.027, *n* = 68, *p* = .828) after partialling out the effects of age and gender.

**Inter-task comparison:** preference for social stimuli across the two tasks: Preferential looking and Button tasks (completed by 49 participants) were not correlated with each other (*r*_s_ = .099, *p = .497*).
